# Transcription and Metabolism Pathways of Anthocyanin in Purple Shamrock (*Oxalis triangularis* A.St.-Hil.)

**DOI:** 10.3390/metabo12121290

**Published:** 2022-12-19

**Authors:** Baobing Luo, Liujun Chen, Guoping Chen, Yunshu Wang, Qiaoli Xie, Xuqing Chen, Zongli Hu

**Affiliations:** 1 Laboratory of Molecular Biology of Tomato, Bioengineering College, Chongqing University, Chongqing 400044, China; 2 Institute of Grassland, Flowers and Ecology, Beijing Academy of Agriculture and Forestry Sciences, Beijing 100097, China

**Keywords:** anthocyanin biosynthesis, *Oxalis triangularis*, UPLC-ESI-MS/MS, RNA-seq, stress resistance

## Abstract

Anthocyanins are water-soluble pigments that can impart various colors to plants. Purple shamrock (*Oxalis triangularis*) possesses unique ornamental value due to its purple leaves. In this study, three anthocyanins, including malvidin 3-O-(4-O-(6-O-malonyl-glucopyranoside)-rhamnopyranosyl)-5-O-(6-O-malonyl-glucopyranoside), delphinidin-3-O-rutinoside and malvidin-3,5-di-O-glucoside, were characterized with ultra-performance liquid chromatography-electrospray ionization tandem mass spectrometry (UPLC-ESI-MS/MS) in purple shamrock. To investigate the molecular mechanism of anthocyanin biosynthesis in green shamrock (*Oxalis corymbosa*) and purple shamrock, RNA-seq and qRT-PCR were performed, and the results showed that most of the anthocyanin biosynthetic and regulatory genes were up-regulated in purple shamrock. Then, dark treatment and low temperature treatment experiments in purple shamrock showed that both light and low temperature can induce the biosynthesis of anthocyanins.

## 1. Introduction

Anthocyanins, a group of water-soluble pigments, are widely spread in higher plants. They are flavonoid compounds derived from the phenylpropanoid biosynthesis pathway [1,2]. While flavonoids, as the most important secondary metabolites in plants, can produce the colors of purple, blue and red in flowers, leaves and fruits [3]. Anthocyanins confer a variety of colors to plants, which contributes to the completion of pollination and seed dispersal via attracting insects or animals [4]. In addition, they also play important roles in resisting biotic and abiotic stress [4], such as cold, drought, salt and UV irradiation. Furthermore, more and more evidence show that intaking foods rich in anthocyanins can effectively reduce the risk of suffering from obesity [4], cancers [5,6], and cardiovascular disease [7,8]. For ornamental plants, cultivars that contain an amount of anthocyanins are more eye-catching than others.

So far, the anthocyanin biosynthetic pathway has been studied extensively in higher plants, including tree peony (*Paeonia suffruticosa Andrews*) [9], tomato (*Solanum lycopersicum* L.) [10], rugosa rose (*Rosa rugosa* Thunb.) [11], and others. Anthocyanin biosynthesis is classified as a branch of the phenylpropanoid pathway, which requires the participation of multiple enzymes. The genes encoding these enzymes can be divided into three categories, namely beginning, early, and late biosynthetic genes [12,13]. The beginning biosynthetic genes consist of PAL, C4H, and 4CL, which are in the phenylpropanoid pathway. The early biosynthetic genes (EBGs), CHS, CHI and F3H, are involved in a common flavonoid pathway. While the late biosynthetic genes (LBGs), including F3′H, F3′5′H, DFR, ANS and UFGT, lead to the production of the different flavonoid pigments in many plants.

Over the past years, many transcription factors (TFs), including MYBs, bHLHs and WDRs, were identified in many plant species [14,15,16]. R2R3-MYBs have been shown to be involved in the anthocyanin biosynthetic pathway via activating the enzyme genes in many plants. Previous studies showed that these TFs can form MYB-bHLH-WD40 (MBW) complex binding to the promoter of enzyme genes and promoting the transcription of these genes [1,17,18]. Recent reports indicated that WRKY TFs are also involved in the regulation of anthocyanin biosynthesis [1,12,19]. In this study, genes coding for six TFs, including MYB113, TT8, TTG1, TTG2, GL3 and CPC, were characterized. Among these genes, CPC, a MYB TF, is a negative regulator of anthocyanin biosynthesis in *Arabidopsis thaliana* L. [20,21].

Environmental conditions, such as light and temperature, also affect anthocyanin biosynthesis in many plants. Light could induce the expression of light-responsive genes, such as HY5 and COP1, which regulate the expression of MYB TF genes that are related to anthocyanin biosynthesis [22,23]. Similarly, low temperature can also induce the expression of low temperature response factor genes to affect the anthocyanin levels in plants. For example, CBFs can interact with MYB113 to regulate anthocyanin biosynthesis in eggplant (*Solanum melongena* L.) [24]. In kiwifruit (*Actinidia chinensis* Planch.), light and low temperature both can induce the expression of AcMYB10, which leads to the increase of anthocyanin content [15]. In contrast, anthocyanin accumulation in kiwifruit decreased under high temperature conditions [25]. An exposure to light induced PpbHLH64 expression and anthocyanin accumulation in pear fruit (*Pyrus pyrifolia* Nakai) [26]. In purple head Chinese cabbage (*Brassica rapa* L.), BrMYB2, and BrTT8 were highly up-regulated after low temperature treatment, which promotes the biosynthesis of anthocyanins [27].

*Oxalis corymbosa* DC., the green shamrock, belongs to the family Oxalidaceae and is widely distributed all over the world. Groom et al. [28] demonstrated that *Oxalis corniculata* probably comes from southeastern Asia. In addition, *Oxalis corniculata* is regarded as an herb with antibacterial, antifungal and anticancer potentials [29,30,31] and has the third largest distribution in vascular plants [32]. *Oxalis triangularis* A. St.-Hil., also called purple shamrock or false shamrock [33], is an important traditional ornamental and medicinal plant. As an ornamental plant, the purple leaves of this plant are the most outstanding feature. The trifoliate leaves are subdivided into three obtriangular to obovate-triangular leaflets and resemble a clover in shape. The leaves of purple shamrock move in response to light levels, opening in high ambient light (in the day) and closing at low light levels (at night).

**Figure 1:**
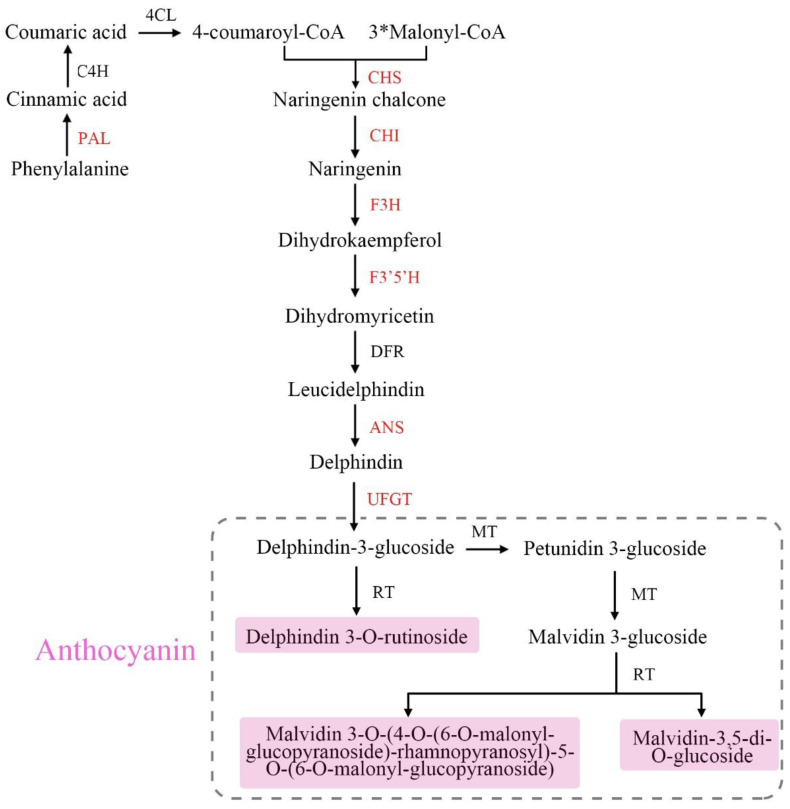
Anthocyanin biosynthetic pathway in purple shamrock. Abbreviations: PAL, phenylalanine ammonia lyase; C4H, cinnamate 4-hydroxylase; 4CL, 4-coumarateCoA ligase; CHS, chalcone synthase; CHI, chalcone isomerase; F3H, flavanone 3-hydroxylase; F3′H, flavonoid 3′-hydroxylase; F3′5′H, flavonoid 3′5′-hydroxylase; DFR, dihydroflavonol 4-reductase; ANS, anthocyanidin synthase; UFGT, flavonoid 3-O-glucosyltransferase; RT, rhamnosyltransferase; MT, methyltransferase.

So far, the molecular mechanisms underlying the different leaf colors between purple and green shamrocks are still unclear. In this study, the anthocyanins in purple shamrock were characterized with ultra-performance liquid chromatography-electrospray ionization tandem mass spectrometry (UPLC-ESI-MS/MS). The anthocyanin biosynthetic and regulatory genes were analyzed by real-time quantitative reverse transcription PCR (qRT-PCR) in the two species of shamrocks. Meanwhile, the expression levels of anthocyanin biosynthetic and regulatory genes were further determined in both species under dark/light conditions. RNA-seq was also used to analyze the differentially expressed genes (DEGs) in the leaves of green and purple shamrocks. Based on the above, the biosynthetic pathway of anthocyanins in purple shamrock was summarized (Figure 1). These results further our understanding about the molecular mechanisms of anthocyanin biosynthesis in purple shamrock.

## 2. Materials and Methods

### 2.1. Plant Materials and Growth Conditions

**Figure 2:**
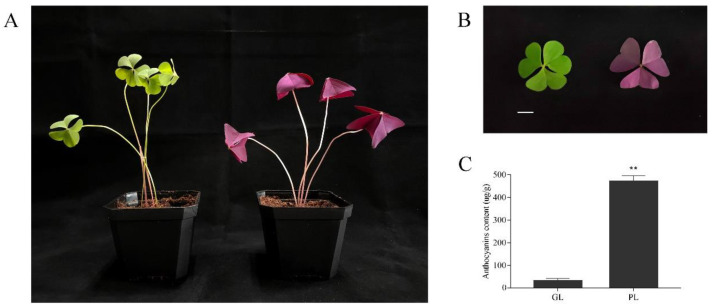
(**A**) Phenotypes of green and purple shamrocks. (**B**) The leaf of green and purple shamrocks. Bar = 1 cm. (**C**) Total contents of anthocyanins in leaves of green and purple shamrocks. Abbreviations: GL, leaves of green shamrock; PL, leaves of purple shamrock. ** indicates a significant difference (*p* < 0.01).

The bulbs of green shamrock (*Oxalis corymbosa* DC.) and purple shamrock (*Oxalis triangularis* A.St.-Hil.) were used as the original experimental materials. The two plants reached maturity (Figure 2A) after 30 days of culture under the following conditions: 28 °C/light for 16 h (intensity of 250 µmol m-2 s-1), 18 °C/dark for 8 h, 80% relative humidity (RH). The mature leaves (Figure 2B) of green and purple shamrock were used for UPLC-ESI-MS/MS analysis. The samples used for qRT-PCR analysis were collected from mature leaves and flowers of green and purple shamrocks. For dark treatment, the purple shamrocks plants were grown in the dark (28 °C/dark for 16 h, 18 °C/dark for 8 h, 80% RH.)/light (28 °C/light for 16 h, 18 °C/dark for 8 h, 80% RH.) conditions, respectively, for 30 days. For low temperature treatment, the purple shamrocks were cultivated in normal (28 °C/light for 16 h, 18 °C/dark for 8 h, 80% RH.)/low (4 °C/light for 16 h, 4 °C/dark for 8 h, 80% RH.) temperature conditions for 0, 3, 6, and 12 days, respectively. All samples were frozen in liquid nitrogen and stored at −80 °C until further analysis.

### 2.2. Extraction and Total Concentration of Anthocyanins

The method of Rapisarda et al. [34] was used to determine the total anthocyanin content in plant tissues. Frozen samples (100 mg) were ground into powder in liquid nitrogen and extracted separately with 1 mL of pH 1.0 buffer (50 mM KCl and 150 mM HCl) and 1 mL of pH 4.5 buffer (400 mM CH3COONa and 240 mM HCl). Then, the mixtures were centrifuged at 14,000× *g* for 5 min at 4 °C. The supernatants were gathered for measurement of absorbance at 510 nm. Concentration of anthocyanins was calculated using the following equation:

concentration (µg g^−1^ FW) = (A1 − A2) × 484.8 × 1000/24,825 × dilution factor

A1 represents the absorbance of supernatants gathered from pH 1.0 buffer solution at 510 nm, while A2 represents the absorbance of supernatants gathered from pH 4.5 buffer solution at 510 nm. The value 484.8 represents the molecular mass of cyanidin-3-glucoside chloride, while 24,825 (L g^−1^ cm^−1^) reflects its molar absorption coefficient at 510 nm in the pH 1.0 solution. Three biological replicates were used for total anthocyanin extraction, and each sample was from a different plant.

### 2.3. UPLC and ESI-MS/MS Analysis of Anthocyanins

The sample extracts were analyzed using an UPLC-ESI-MS/MS system (UPLC, Shim-pack UFLC SHIMADZU CBM30A system; MS, Applied Biosystems 4500 Q TRAP). The analytical conditions were as follows, UPLC: column, Agilent SB-C18 (1.8 µm, 2.1 mm × 100 mm); The mobile phase consisted of solvent A, pure water with 0.1% formic acid, and solvent B, acetonitrile. Sample measurements were performed with a gradient program that employed the starting conditions of 95% A, 5% B. Within 9 min, a linear gradient to 5% A, 95% B was programmed, and a composition of 5% A, 95% B was kept for 1 min. Subsequently, a composition of 95% A, 5.0% B was adjusted within 1.1 min and kept for 2.9 min. The column oven was set to 40 °C; the injection volume was 4 μL. The effluent was alternatively connected to an ESI-triple quadrupole-linear ion trap (QTRAP)-M.

The ESI source operation parameters were as follows: ion source, turbo spray; source temperature 550 °C; ion spray voltage (IS) 5500 V (positive ion mode)/−4500 V (negative ion mode); ion source gas I (GSI), gas II (GSII), curtain gas (CUR) was set at 50, 60, and 30.0 psi, respectively; the collision gas (CAD) was high. Instrument tuning and mass calibration were performed with 10 and 100 μmol/L polypropylene glycol solutions in QQQ and LIT modes, respectively. QQQ scans were acquired as MRM experiments with collision gas (nitrogen) set to 5 psi. DP and CE for individual MRM transitions was performed with further DP and CE optimization. A specific set of MRM transitions were monitored for each period according to the metabolites eluted within this period. Three biological replicates were used for UPLC and ESI-MS/MS analysis, and each sample was from a different plant.

### 2.4. RNA Extraction and qRT-PCR Analysis

The frozen samples were crushed into powder in liquid nitrogen. Total RNA was isolated from the samples with RNAiso (Takara, Otsu, Japan), and 1–2 µL of total RNA was used to synthesize the complementary DNA (cDNA) with reverse transcriptase (Promega, Beijing, China) and an oligo (dT)20 primer. The qRT-PCR was carried out using the CFX96 real-time system (Bio-Rad, Hercules, CA, USA). Three biological replicates were used for qRT-PCR analysis, and each sample was from a different plant. The primers used for qRT-PCR analysis were designed by Primer Premier 5 (Table S1).

### 2.5. Library Preparation and Transcriptome Sequencing

Total RNA was used as input material for the RNA sample preparations. Sequencing libraries were generated using NEBNext^®^ Ultra™ RNA Library Prep Kit for Illumina^®^ (NEB, Waltham, MA, USA) following the manufacturer’s recommendations. Briefly, mRNA was purified from total RNA using oligo (dT)-attached magnetic beads. The first strand cDNA was synthesized using random hexamer primer and M-MLV Reverse Transcriptase. Second strand cDNA synthesis was subsequently performed using DNA Polymerase I. The cDNA fragments were end-repaired and ligated to NEBNext adaptor. Then, the library fragments were purified with AMPure XP system to select cDNA fragments of preferentially 250–300 bp in length, and PCR was performed to enrich the cDNA fragments. Finally, the library preparations were sequenced on an Illumina Hiseq platform and paired-end reads were generated. Three biological replicates were used for RNA-seq analysis, and each sample was from a different plant.

### 2.6. Establishment of Local mRNA and Protein Database

Since there is no genome information of *Oxalis triangularis* online, the result of RNA-seq analysis was used to establish the local mRNA and protein database for further research. BioEdit, a sequence analysis software, was used to build the database by importing mRNA and protein sequence information. Based on a BLASTP search using the amino acid sequences of anthocyanin biosynthetic genes in Arabidopsis as queries, single orthologs for PAL, CHS, CHI, F3H, F3′5′H, ANS, and UFGT were obtained from purple shamrock contigs. In addition, the regulatory genes, including MYB113, TT8, TTG1, TTG2, GL3 and CPC, were also identified.

### 2.7. Differential Expression Analysis

Differential expression analysis of the two groups was performed using the DESeq R package (1.10.1). DESeq provides statistical routines for determining differential expression in digital gene expression data using a model based on the negative binomial distribution. The resulting P values were adjusted using the Benjamini and Hochberg’s approach for controlling the false discovery rate [35]. Genes with an adjusted *p*-value < 0.05 found by DESeq were assigned as differentially expressed.

### 2.8. Gene Functional Annotation and Enrichment Analysis

To acquire comprehensive gene function information, seven databases, including Nr (NCBI non-redundant protein sequences), Nt (NCBI non-redundant nucleotide sequences), Pfam (Protein family), KOG/COG (Clusters of Orthologous Groups of proteins), Swiss-Prot (A manually annotated and reviewed protein sequence database), KO (KEGG Ortholog database) and GO (Gene Ontology), were used to annotate the assembled unigenes. Expression level of each transcript was estimated by FPKM (fragments per kilobase of transcript per million fragments mapped) method [36].

## 3. Results

### 3.1. Identification of Anthocyanins in Green and Purple shamrocks

**Figure 3:**
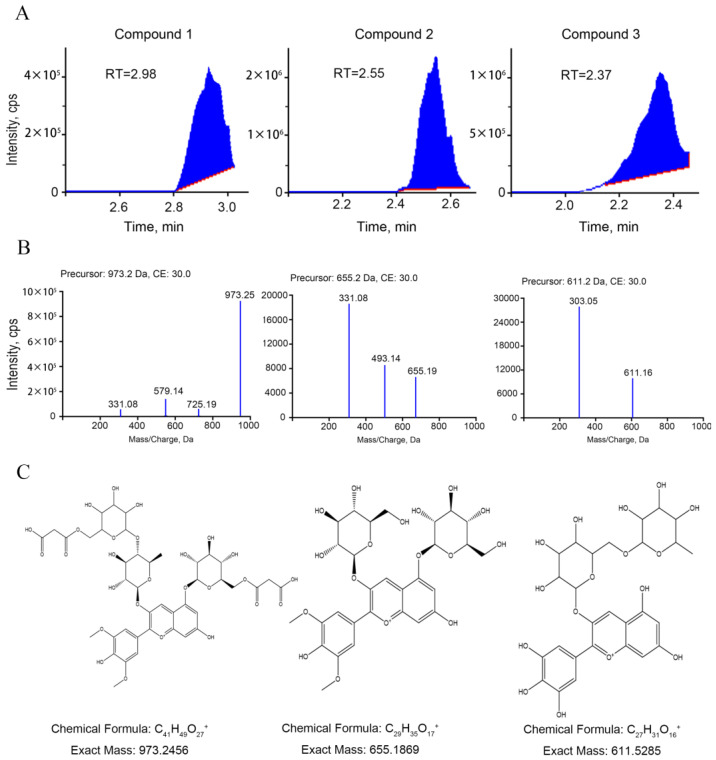
UPLC and ESI-MS/MS Analysis of Anthocyanins. (**A**) Chromatograms of three anthocyanins detected in green and purple shamrocks. (**B**) Mass spectrogram of three anthocyanins. (**C**) Structural formulas and chemical formulas of three anthocyanins.

By analyzing the extracts from leaves of green and purple shamrocks with the method of UPLC-ESI-MS/MS, three anthocyanins were separated and identified (Figure 3). As shown in Table 1, three anthocyanins, including malvidin 3-O-(4-O-(6-O-malonyl-glucopyranoside)-rhamnopyranosyl)-5-O-(6-O-malonyl-glucopyranoside), delphinidin-3-O-rutinoside and malvidin-3,5-di-O-glucoside, were characterized. Through KEGG enrichment analysis, these three anthocyanins are derivatives of delphinidin-3-O-glucoside that is the main anthocyanin for purple plants (Figure S1). To further verify the effect of anthocyanins on purple shamrock’s phenotype, total content of anthocyanins was determined. As expected, the total content of anthocyanins in purple shamrock was about 15 times that of green shamrock (Figure 2C).

### 3.2. Transcriptome Analysis of Green and Purple Shamrocks by RNA-seq

The mature leaf samples of green and purple shamrocks were collected for RNA-seq analysis to investigate the molecular mechanisms of anthocyanin accumulation in purple leaves. To identify the differentially expressed genes (DEGs) involved in purple leaf coloration, the fragment per kilobase of exon per million fragments mapped (FPKM) values were analyzed for each gene in leaves of green and purple shamrocks.

**Figure 4:**
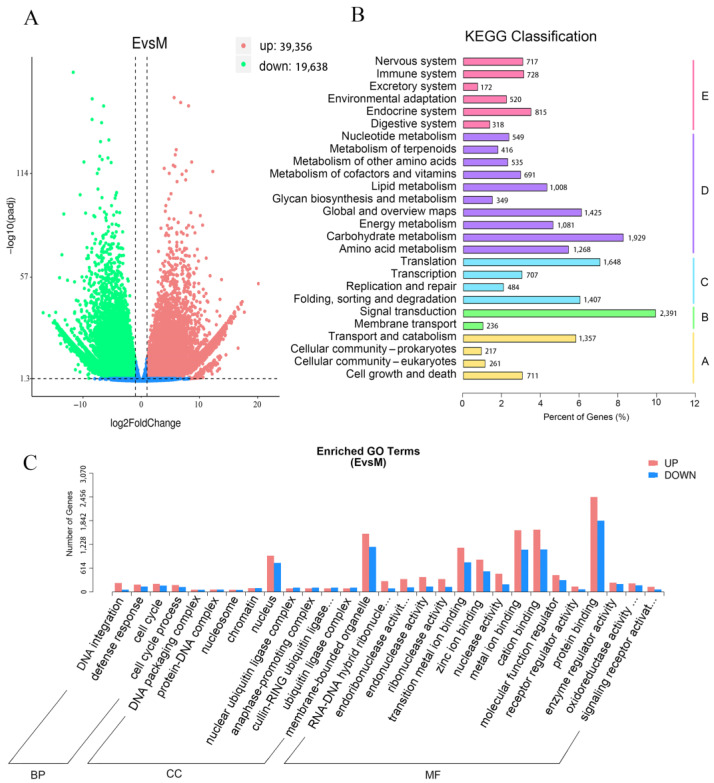
DEGs, KEGG classification and GO Enrichment analysis. (**A**) Volcano plot of differentially expressed genes. (**B**) KEGG classification of assembled differentially expressed genes. Abbreviations: A, cellular processes; B, environmental information processing; C, genetic information processing; D, metabolism; E, organismal systems. (**C**) GO Enrichment of assembled differentially expressed genes. Abbreviations: BP, biological processes; CC, cellular components; MF, molecular functions.

As shown in the volcanic plot map, a total of 39,356 DEGs were upregulated and 19,638 DEGs were downregulated (Figure 4A). To classify the genes involved in the anthocyanin biosynthetic pathway, all DEGs were subjected to KEGG database. According to the different functions, the DEGs were divided into five categories and thirty-four subcategories. The five categories including cellular processes, environmental information processing, genetic information processing, and metabolism and organismal systems, and the DEGs mainly belong to metabolism. (Figure 4B). For further research, GO enrichment was performed. All of the DEGs based on their functions were divided into three main categories, including four biological processes (BP) terms; 10 cellular components (CC) terms and 15 molecular function (MF) terms. For CC, the majority of DEGs were associated with nuclear and membrane-bound organelle functions. For MF, most of DEGs were related to binding and catalytic activity (Figure 4C). Above all, although there are many DEGs between green and purple shamrocks, only a small part of them are involved in anthocyanin biosynthesis (Supporting Information 1, Supporting Information 2 and Figure S5).

### 3.3. Expression of Anthocyanin Biosynthetic and Regulatory Genes in Green and Purple Shamrocks

**Figure 5:**
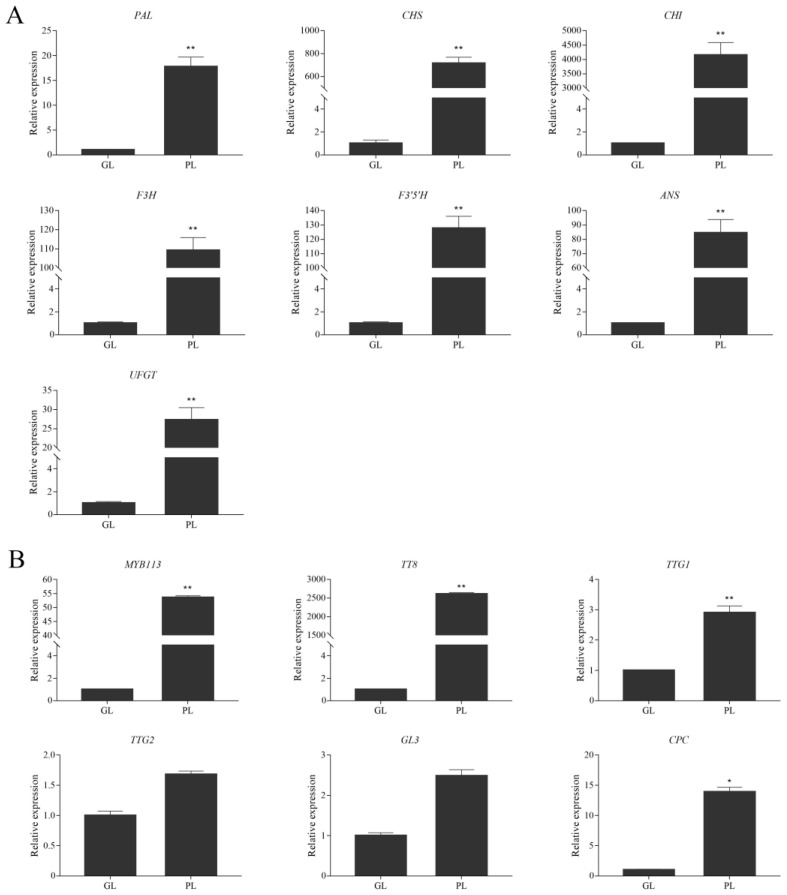
Relative expression level analysis of anthocyanin biosynthetic and regulatory genes in leaves of green and purple shamrocks. (**A**) Relative expression level analysis of anthocyanin biosynthetic genes. (**B**) Relative expression level analysis of regulatory genes. Abbreviations: GL, leaves of green shamrock; PL, leaves of purple shamrock. Error bars represent the standard error of the mean (*n* = 3). Significant differences (* *p* < 0.05, ** *p* < 0.01).

Through qRT-PCR technology, the expression levels of anthocyanin biosynthetic genes PAL, CHS, CHI, F3H, F3′5′H, ANS and UFGT were analyzed. Compared with the green shamrock, all the anthocyanin pathway genes were significantly upregulated in purple shamrock, which is consistent with the results of RNA-seq (Figure S2). Moreover, the expression levels of CHS and CHI were especially increased at least 700-fold in the purple as compared with the green shamrock (Figure 5A). Meanwhile, the transcripts of some anthocyanin biosynthesis regulatory orthologous genes of Arabidopsis, MYB113, TT8, TTG1, TTG2, GL3 and CPC, were detected in shamrocks. As shown in Figure 5B, all the regulatory genes were upregulated in purple shamrock, especially MYB113 and TT8. These results indicated that the expression of anthocyanin pathway genes in purple shamrock is activated.

### 3.4. Effects of Dark Treatment on Anthocyanin Accumulation in Leaves of Purple Shamrock

**Figure 6:**
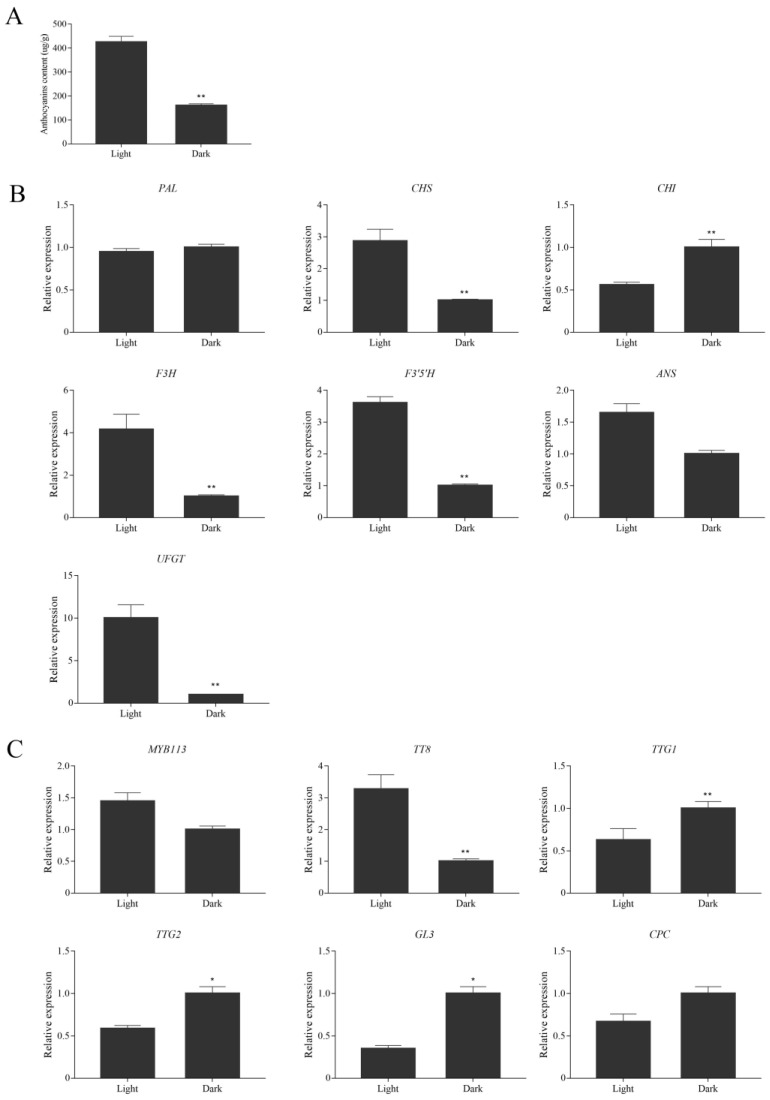
Relative expression level analysis of anthocyanin biosynthetic and regulatory genes in purple shamrock under light/dark conditions. (**A**) Total contents of anthocyanins in light/dark conditions of purple shamrock. (**B**) Relative expression level analysis of anthocyanin biosynthetic genes. (**C**) Relative expression level analysis of regulatory genes. Error bars represent the standard error of the mean (*n* = 3). Significant differences (* *p* < 0.05, ** *p* < 0.01).

According to previous reports [22,23], light could induce anthocyanin accumulation in plants. In this study, purple shamrocks were cultured for 30 days under light and dark conditions. After 30 days of dark treatment, the leaf area of purple shamrocks was much smaller than that grown under normal light, and the color changed to pale pink (Figure S4). In addition, the stem became slender and easy to lodge. The anthocyanin content of purple shamrock leaves grown under dark condition was significantly less than that under light condition (Figure 6A). Further, the expression of anthocyanin biosynthetic and regulatory genes was analyzed by qRT-PCR. As shown in Figure 6B, most of the anthocyanin biosynthetic genes in purple shamrock under dark condition were downregulated compared to that under light condition except PAL and CHI. Moreover, the expression level of CHI was significantly up-regulated after dark treatment, indicating that expression of OtCHI may not be induced by light. Meanwhile, MYB113 and TT8, the key regulators of anthocyanin biosynthesis, were downregulated under dark condition (Figure 6C), although there are no obvious changes in other regulatory genes. These results showed that dark treatment inhibits the expression of some anthocyanin biosynthetic and regulatory genes, thereby reducing the accumulation of anthocyanins in purple shamrock.

### 3.5. Effect of Low Temperature Treatment on Anthocyanin Accumulation in Leaves of Purple Shamrock

**Figure 7:**
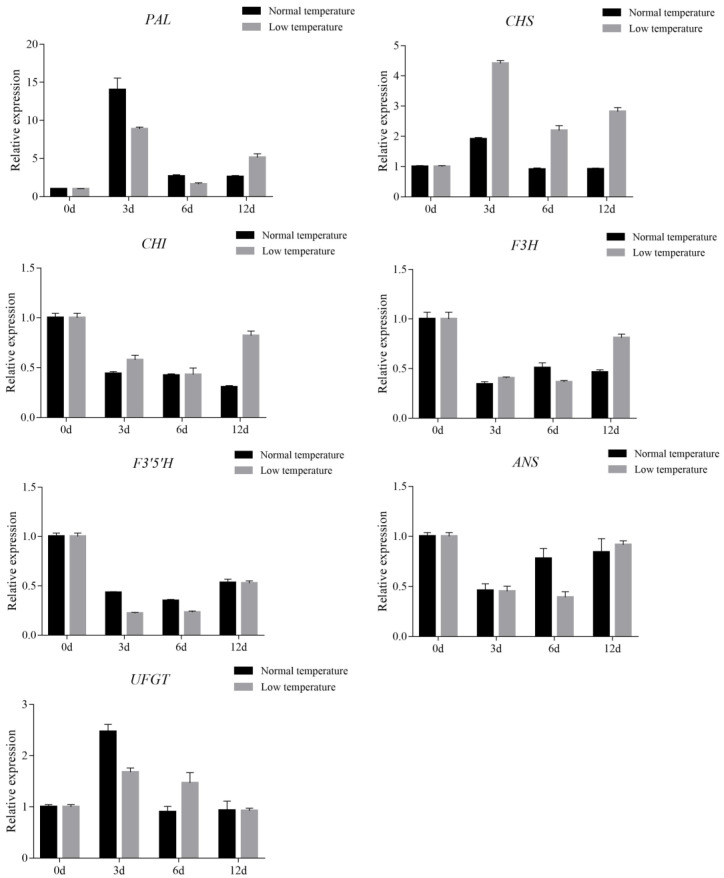
Relative expression level analysis of anthocyanin biosynthetic genes in purple shamrock under normal/low temperature conditions. Error bars represent the standard error of the mean (*n* = 3).

Previous studies showed that temperature can affect the biosynthesis of anthocyanins [27,37]. In this study, purple shamrocks were cultured at 4 °C and normal temperature for 0, 3, 6, and 12 days. After 12 days of low temperature treatment, the color of the purple shamrock leaves is a little darker than that growth under normal temperature, and the anthocyanin content of leaves after low temperature treatment is higher (Figure S3). To further reveal the molecular mechanism of the effect of low temperature on anthocyanin biosynthesis, the expression levels of anthocyanin biosynthetic and regulatory genes were analyzed by qRT-PCR. The results showed that the transcription level of CHS in low temperatures was significantly higher than that in normal temperatures, but there were no significant differences in other anthocyanin biosynthetic genes (Figure 7).

**Figure 8:**
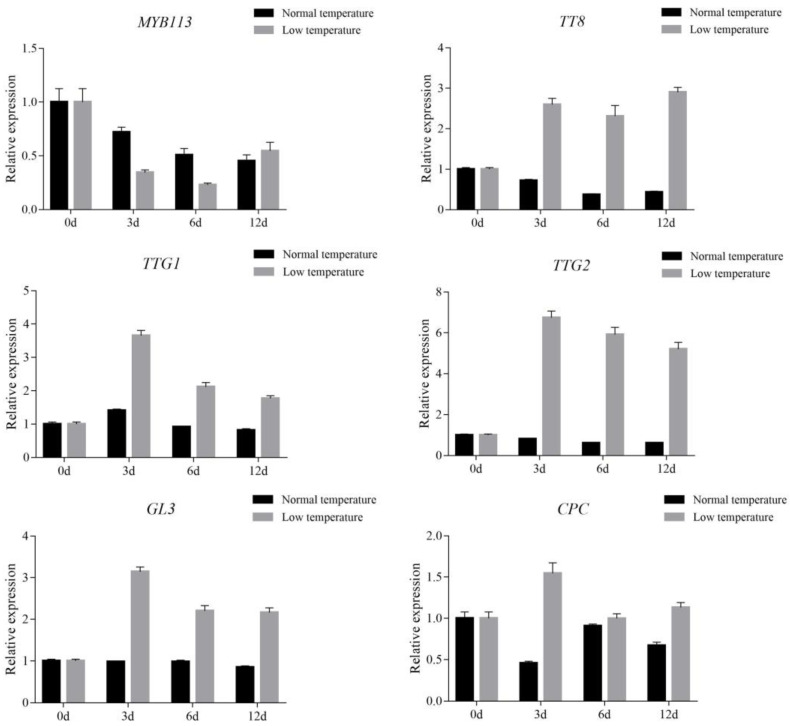
Relative expression level analysis of regulatory genes in purple shamrocks under normal/low temperature conditions. Error bars represent the standard error of the mean (*n* = 3).

Moreover, most of the regulatory genes were upregulated in purple shamrock leaves exposure to low temperature, except MYB113 (Figure 8). These results indicated that low temperature treatment increases the accumulation of anthocyanins in purple shamrock leaves by promoting the expression of some anthocyanin biosynthetic and regulatory genes.

### 3.6. Expression Levels of Anthocyanin Biosynthetic and Regulatory Genes in Flowers of Green and Purple Shamrocks

**Figure 9:**
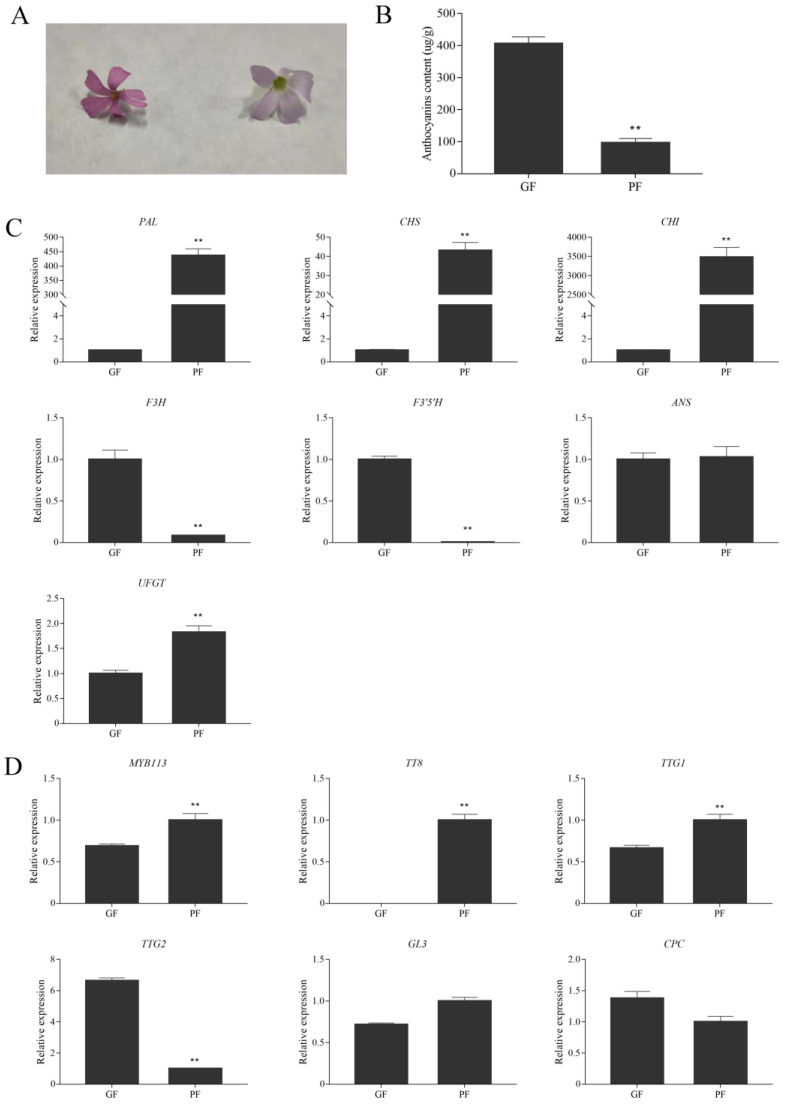
Relative expression level analysis of anthocyanin biosynthetic and regulatory genes in flowers of green and purple shamrocks. (**A**) The flower of green (left) and purple (right) cultivars. (**B**) Total contents of anthocyanins in flowers of green and purple shamrocks. (**C**) Relative expression level of anthocyanin biosynthetic genes in flowers of green and purple shamrocks. (**D**) Relative expression level of regulatory genes in flowers of green and purple shamrocks. Abbreviations: GF, flowers of green shamrock; PF, flowers of purple shamrock. Error bars represent the standard error of the mean (*n* = 3). Significant differences (** *p* < 0.01).

Interestingly, the flowers of green shamrock are darker in appearance than those of purple shamrock (Figure 9A). As shown in Figure 9B, the anthocyanin content in flowers of purple shamrock was markedly lower than that of green shamrock. To investigate the molecular mechanism of anthocyanin accumulation in flowers, qRT-PCR was performed. In flowers of green shamrock, just one regulatory gene, TTG2, has a higher expression level than in purple shamrock (Figure 9D). Besides, among the anthocyanin biosynthetic genes, only F3H and F3′5′H were upregulated in green shamrock (Figure 9C). According to the results mentioned above, in the flowers and leaves of shamrocks, anthocyanin biosynthesis may have different regulatory mechanisms.

## 4. Discussion

Anthocyanins are flavonoid compounds found in many plants, which is the main reason why so many plants have various colors, ranging from red to blue [38]. The biosynthesis of anthocyanins is affected by many factors; therefore, its regulation is quite complex. The color of purple shamrock leaves is the most charming feature. However, as an ornamental plant, this species has not been investigated at the molecular level. To investigate the molecular mechanisms of anthocyanin biosynthesis, UPLC-ESI-MS/MS was performed first to analyze the types of anthocyanins in purple shamrock, and three anthocyanins were identified. According to KEGG pathway, interestingly, all these three anthocyanins are derivatives of delphinidin-3-O-glucoside that makes plants appear purple [39]. Consistently, F3′5′H is highly expressed in purple shamrock, which commits the pathway to delphinidin biosynthesis. As reported by Fossen et al. [40], seven identified anthocyanins also belonged to delphinidin-type anthocyanins, consistent with our results. Anthocyanin biosynthesis is an enzyme cascade reaction process. In this pathway, all anthocyanin biosynthesis genes in purple shamrock that were identified are significantly up-regulated in comparison with green shamrock. This result may be caused by inhibition of the upstream genes of anthocyanin biosynthesis in green shamrock.

In plants, anthocyanins are compartmented in specific locations. For example, stable anthocyanins formed by different modifications, such as glycosylation, methylation and acylation, are transported to the vacuole where they accumulate [41]. Anthocyanins function in plants as protection against environmental stresses, including light, cold, salt and drought stresses [42]. Light is a key environmental factor that can affect anthocyanin biosynthesis in many plants. In litchi (*Litchi chinensis* Sonn.), anthocyanins accumulated rapidly in the pericarp under light condition [43]. The same phenomenon also occurs in teinturier grape (*Vitis vinifera* L.) [44], apple (*Malus domestica* Borkh.) [45], and pear [46]. In this report, the anthocyanin content in the leaves of purple shamrock significantly decreased after dark treatment. Under dark conditions, although the anthocyanin biosynthesis was inhibited, there was still a small amount of anthocyanin accumulation, which indicated that anthocyanin biosynthesis in purple shamrock has two ways: light-dependent and light-independent. However, the molecular mechanism of light-independent anthocyanin biosynthesis remains unclear. Temperature also affects the biosynthesis of anthocyanins. High temperature inhibits anthocyanin biosynthesis, e.g., in Cymbidium hybrids [47], while low temperature induces anthocyanin biosynthesis, as in blood oranges (*Citrus sinensis* L. Osbeck) [37], and Arabidopsis [48]. According to the qRT-PCR results, the expression of CHS was upregulated after low temperature treatment, which is consistent with Naing’s report [49]. Meanwhile, most of the regulatory genes were significantly upregulated in low temperature, except for MYB113, suggesting that MYB113 in purple shamrock may not be affected by low temperature.

Interestingly, during the research, we found that flowers of green shamrock contain more anthocyanins than flowers of purple shamrock. In flowers of green shamrock, the expression of F3H, F3′5′H, was higher than that of flowers of purple shamrock, while the expression of PAL, CHS, CHI, was evidently lower. It is worth noting that TT8 is almost not expressed, while TTG2 is highly expressed in flowers of green shamrock. This result indicated that the biosynthesis of anthocyanins in flowers may be regulated by another mechanism. For example, TTG2 and ARF8 control flower coloration by regulating anthocyanin biosynthesis in tobacco (*Nicotiana tabacum* L.) [50]. According to previous studies, DFR can catalyze the conversion of dihydroquercetin to leucoanthocyanidins. However, FLS catalyzes dihydroflavonols to flavonols, which competes with DFR in the anthocyanin pathway at a key branch point [51]. In tea (*Camellia sinensis* L.), three DFR gene variants were upregulated in pink flowers relative to white flowers. On the contrary, FLS has a higher level of expression in white flowers [52]. Above all, although the key genes for anthocyanin biosynthesis have been identified, there are still many potential genes involved in anthocyanin biosynthesis.

In summary, this research clarified the molecular mechanism of anthocyanin biosynthesis in purple shamrock and explored the response of anthocyanin biosynthesis under different external environmental conditions, such as light and low temperature. Meanwhile, the anthocyanin biosynthesis mechanism of the flowers of the two species was studied, which is in contrast with the anthocyanin biosynthesis in leaves. These results expand our understanding about the molecular mechanism of anthocyanin biosynthesis in purple shamrock.

## Figures and Tables

**Table 1:** Anthocyanin Contents in Leaves of Green and Purple Shamrock.

NO.	Compound	Sample	
		Green	Purple
1	Malvidin 3-O-(4-O-(6-O-malonyl-glucopyranoside)-rhamnopyranosyl)-5-O-(6-O-malonyl-glucopyranoside)	9	4,290,000
2	malvidin-3,5-di-O-glucoside (Malvin)	856,000	22,200,000
3	delphinidin-3-O-rutinoside	416,000	15,000,000

Note: the values in the table represent the area of the chromatographic peak (cps min).

## Data Availability

The data presented in this study are available in the main article and the supplementary materials.

## Supplementary Materials

The following supporting information can be downloaded at: https://www.mdpi.com/article/10.3390/metabo12121290/s1, Table S1: The primers used for qRT-PCR analysis; Figure S1: KEGG enrichment analysis; Figure S2: The expression of anthocyanin pathway genes in RNA-seq; Figure S3: The anthocyanin content of leaves after low temperature treatment; Figure S4: The phenotype of purple shamrock after dark treatment; Figure S5: DEGs in Flavonoid Biosynthesis; Supporting Information 1: Expression levels of genes involved in anthocyanin biosynthesis in the transcriptome; Supporting Information 2: DEGs involved in anthocyanin biosynthesis between green and purple shamrocks.
