# Supplementary material for: Transcription and Metabolism Pathways of Anthocyanin in Purple Shamrock (Oxalis triangularis A.St.-Hil.)
Source: Metabolites. 2022 Dec 19;12(12):1290. doi: 10.3390/metabo12121290 (PMC9784199; doi:10.3390/metabo12121290)
Supplement: Supplementary file 1 [file metabolites-12-01290-s001.zip › Supplementary data.pdf]

gene	sequence
PAL	F: TGGTGGCGGAGTATAGGAAAT<br>R: TGGCACCAAATCCAGTCGTA
CHS	F: TTTCCGTATGTGCAATGCTGA<br>R: CTTAGGAACCTCCTCGACCATAA
CHI	F: ATGATGTTGCCGCTGACTGG<br>R: GAATCGAAGAGCCTGGAGGAA
F3H	F: GCCTCCAAGCCACTAAAGATAA<br>R: GCGATTGATAATCGGCTGTAA
F3'5'H	F: ACTACATCCCAAAGAACTCACGG<br>R: CCGCTCCAAACGGTATCAGT
ANS	F: TACGGAGGCCACAAGTGAGT<br>R: TCGACTCCTAAGGCGAGGT
UFGT	F: CAGGTATGGTGCTAAACGAGG<br>R: TCAACATTGGACCGACAGG
MYB113	F: ATAAGGAAAGGAGCGTGGACTG<br>R: GGCTTCAAGTAGTTCAACCACCT
TT8	F: ACAATGAAATCTCACCCAAGTCC<br>R: ATTTTCGTCACAAATGGGACTAA
TTG1	F: TAGAGTTTTCGCTTCGGTTTCG<br>R: ATGTATCTCAAATCCTGCTTGTTCC
TTG2	F: CTTCTTACGATGGCTACAACTGG<br>R: CGCAATCTGTCCATCTAACGA
GL3	F: GCAATCAGTAAACAAGAGAAAGGC<br>R: GAACCGTGTGACAATCTAAACGTAG
CPC	F： GATGTTCGGAAGAAGTGAGCAGT<br>R： CTACCTGGAATCCGTCCTGCT
Actin	F： ATTTCCCGCTCTGCTGTGG<br>R： AGGTATCGTGCTTGATTCTGGTG

Table S1. The primers used for qRT-PCR analysis.

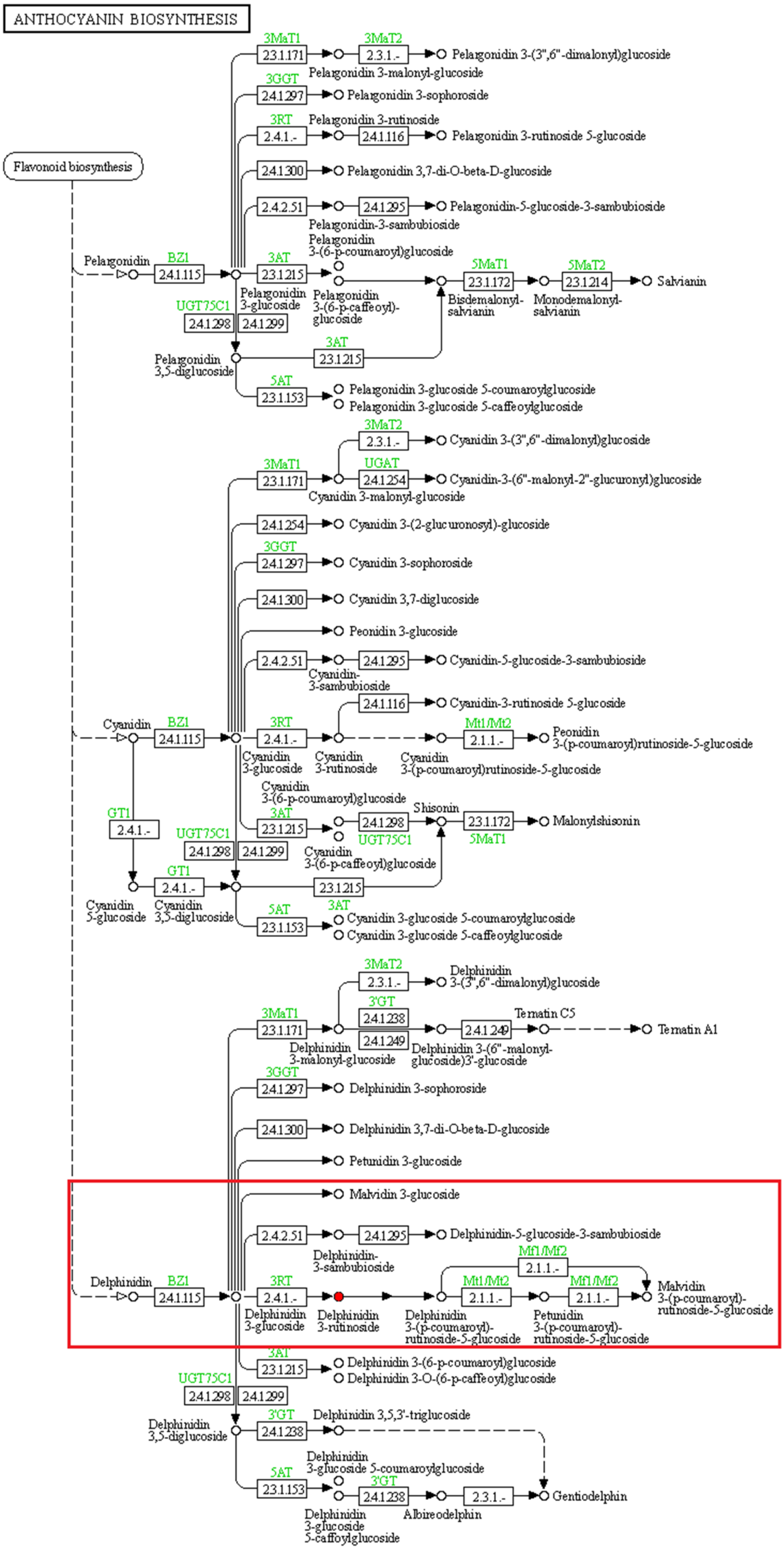

Figure S1. KEGG enrichment analysis.

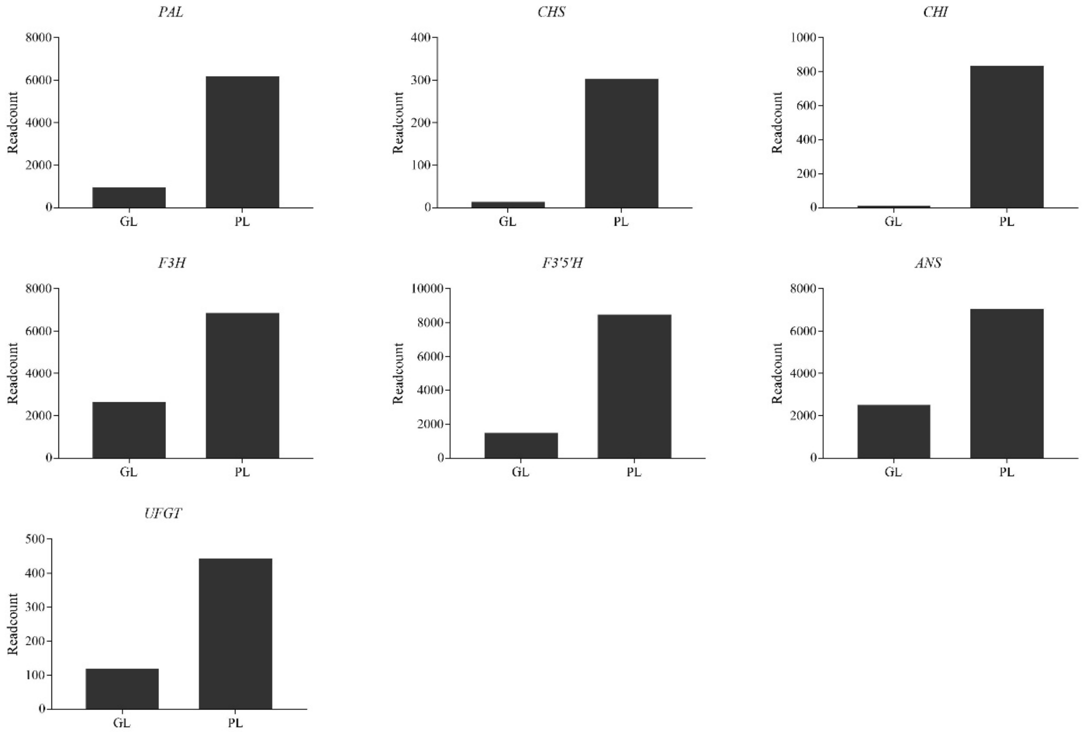

Figure S2. The expression of anthocyanin pathway genes in RNA-seq.

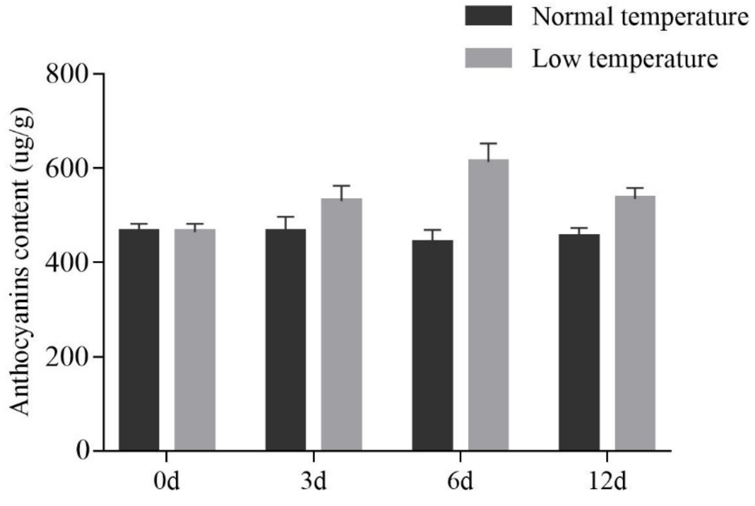

Figure S3. The anthocyanin content of leaves after low temperature treatment.

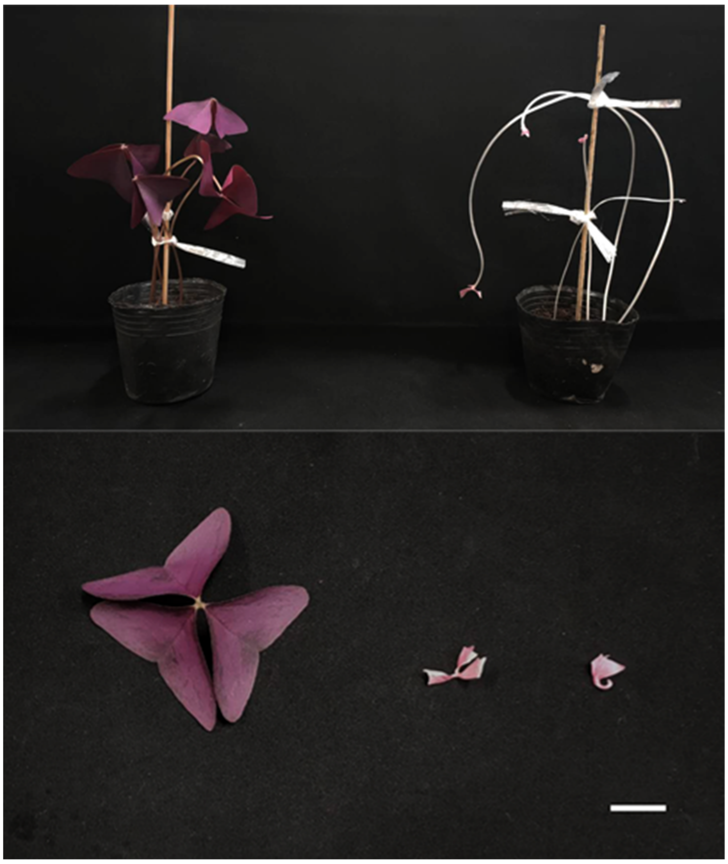

Figure S4. The phenotype of purple shamrock after dark treatment.

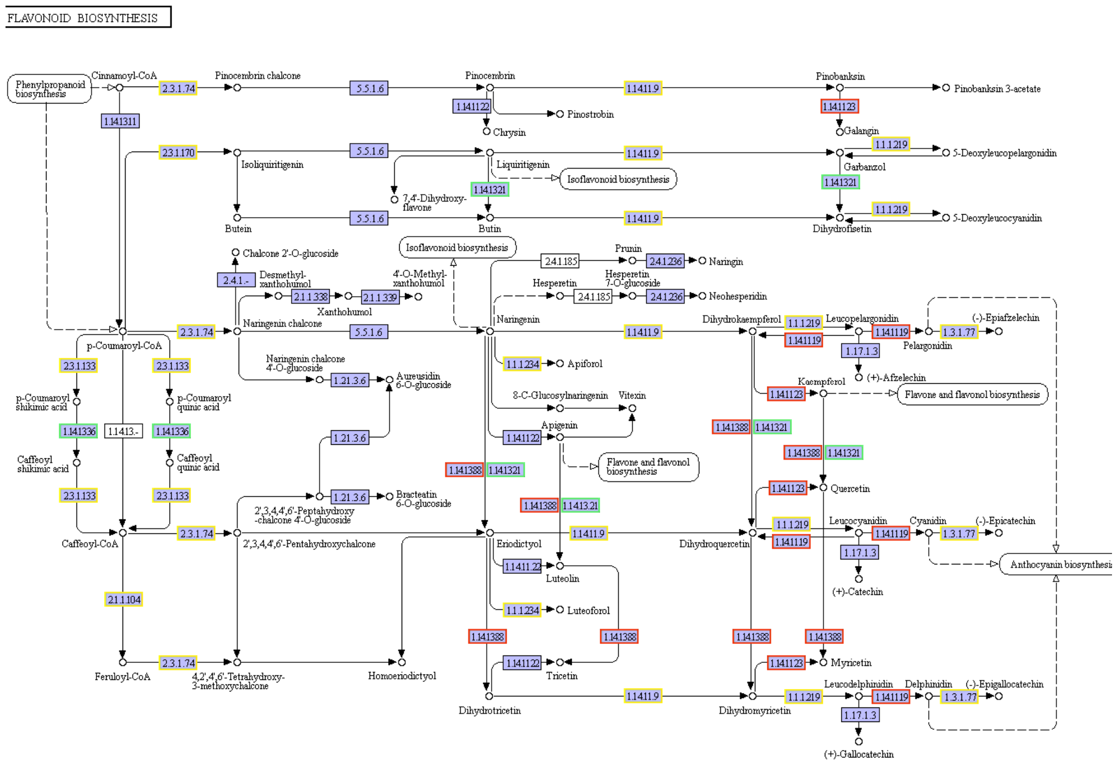

Figure S5. DEGs in Flavonoid Biosynthesis.
